# The League of Mercy

**Published:** 1906-12-22

**Authors:** 


### THE LEAGUE OF MERCY.

### ANNUAL MEETING OF PRESIDENTS.

The seventh annual meeting of Presidents of the League of Mercy was held in the Clarence Memorial Wing of St. Mary's Hospital, Paddington, on December 11, Mr. Frederick Green presiding. Among the many ladies present were the Princesses Victoria and Louise Augusta of Schleswig-Holstein.

In opening the proceedings the Chairman said it was his sad duty to remind them of the death of the Earl of Mansfield, who, as Chairman of the meeting of Presidents, was most efficient and of great value. He had worked hard in the service of the League and did much for its purposes. They all greatly regretted the loss the League had sustained by his death. As to the past year it was satisfactory to be able to report that they had again made steady progress. He would ask Mr. Wallington to read a letter from their Grand President the Prince of Wales.

Mr. Wallington (the Hon. Registrar of the Order) said : Your Highness, Mr. Chairman, ladies and gentlemen :— I am directed by the Prince of Wales to say that the Princess and he share with the Presidents of the League their regret at the untimely death of the Chairman of the Presidents' meeting, the late Lord Mansfield. Last year, owing to his absence in India, His Royal Highness was unable to communicate with the Presidents on the occasion of their annual meeting. He desires now to offer congratulations from the Lady Grand President, as well as from himself, to them and to all those working under them, on the continued progress of the League's operations alike in value and extent. His Royal Highness is glad to learn that the League will in all probability be able to hand over about £17,000 to the King's Hospital Fund. This is the largest amount which has yet been contributed; and it is most gratifying to His Royal Highness to know that the work done by members of the League has served to render such valuable aid, not only to the London hospitals, but also to the various local hospitals in the Home Counties. (Applause.)

Lord Farquhar having been appointed Chairman, Mr. Green, Deputy-Chairman, and Colonel H. B. Hamilton, Hon. Secretary, respectively, for the annual meeting of Presidents for the ensuing year, the following seven representative members of the Executive Committee were appointed—namely, Lord Farquhar, G.C.V.O., Mr. R. C. Antrobus, J.P., Mr. Frederick Green, Mrs. R. L. Harrison, Mrs. Buxton Morrish, Mr. Montagu Sharpe, D.L. (retiring members), and Mr. Edward Murray Ind, D.L., J.P., in the place of Lord Mansfield, deceased.

### Work in the Home Counties.

Mrs. Murdoch, as Hon. Secretary to Princess Victoria of Schleswig-Holstein, gave a short report of the work which has been accomplished under her Highness's presidency in the four counties of Berks, Bucks, Hants, and Oxon.

With regard to *Berkshire*, which was first organised in 1905, the county is practically arranged, and the result this year is £664 10s. 6d. for the League. In addition to this sum the Princess has given another £240 to the four county hospitals—namely, £60 to each, from the Fancy Fair held at Buckhurst in July last, under the presidency of Prince and Princess Christian, which Princess Victoria, with Princess Louise of Schleswig-Holstein, also attended. The following three counties have been in course of organisation during 1906.

In *Hampshire*. The Princess has received great help from Sir William and Lady Portal and the other lady vice-presidents, and the county has now a lady vice-president in each Petty Sessional Division. Although some of the ladies only began working about four months ago the large sum of £469 1s. 2d. has been collected. Very influential meetings have been held at Heron Court, where Lord and Lady Malmesbury have taken the greatest interest in the working of the League. Another meeting has been held at Pylewell Park, where Mr. and the Hon. Mrs. Ingham Whitaker received their guests with the greatest hospitality. These meetings were invaluable, for after they were over, in consequence of the speeches made, many said they wished to join, as they understood so much better what the Princess was pleading for. Another meeting will be held on December 18 at Lady Normanton's, and later on at the Hon. Mrs. Eliot Yorke's at Netley, and Mrs. E. Prideaux-Brune also hopes to have two meetings in her division.

In *Oxfordshire* the Princess has received much thoughtful kindness. Two meetings were held at Blenheim, the first, which was presided over by the Duke of Marlborough, was a very large and influential one. Lady Dillon has also given very valuable help in the organisation, and hopes later on to have a meeting at Ditchley. Large meetings have also been held during the summer and autumn at Lady Algernon Gordon Lennox's, at Broughton Castle, and also at Tythrop House, where Mrs. Wyckham received many guests. Although in Oxfordshire there are many poor and scattered districts, the large sum of £288 2s. 4d. has been given.

In *Buckinghamshire* the Princess thanks Sir William and Lady Clayton for their great help. A very representative gathering took place in this county at Mrs. Lee's, of Hartwell House, which greatly increased the members of the League. The total from that county is £244 16s. Princess Victoria has this year visited the Royal Bucks Hospital at Aylesbury, and the Royal County Hospital at Winchester, where her kindly interest and gracious sympathy won all hearts. She has also graciously promised to visit the Radcliffe Infirmary at Oxford during 1907. It is hoped all those who have worked so unselfishly to further the objects of this League will continue their efforts during 1907.

The Rev. C. H. Ditchfield, M.A., said that with a view to assisting the work of the League he proposed to publish a small book to be called "The Book of the League." It was clear that the League of Mercy had won a place in the hearts of the authors and artists of the United Kingdom, and already he had a brilliant array of contributors.

### The Treasurer's Statement for the Year.

Sir Henry Burdett, the Hon. Treasurer of the League, then made his annual statement. He said it fell to his lot in the course of the year to attend many meetings, but none so full of interest as that. Being held somewhat earlier than usual, the returns had not all been yet received. They had now a total of 100 districts, and of these they had money returned from 68, though some of these were incomplete. Thirty-seven of the districts showed an increased return of £1,803, while 31 showed a decrease of £1,030, a net gain for the year so far of £773. Fulham again headed the list with £1,256, against £1,181 last year, an advance of £75. South Kensington came next with £1,214, as compared with £1,272, but as the return was not complete he had no doubt that last year's total would be beaten. South Paddington, with £705, as against £983, showed a drop of £278, but that was no doubt accounted for by a change of President and Hon. Secretary. The new officers required time to get a grasp of their work, and they were showing a commendable amount of zeal. As a result of the work of Her Highness Princess Victoria, Hampshire showed an advance of £396. Dealing with the smaller amounts, and coming down to the City of London, he repeated the wish he had expressed last year—that someone would take hold of the city and organise it for the League. He was sure there was a great field there. One of the pleasantest features he had to notice was the growth of the country districts. In the home counties especially they had been so organised that they showed striking progress in their results every year. And the total result was £1,666, as compared with £1,047 in 1895. Everyone connected with the League and with hospitals would welcome the signs of growing interest in the home counties in the work of the hospitals. He would also like to refer to the admirable results achieved by the Fulham district. In 1899 they collected £225; this year the amount was £1,257, and the total for the eight years since its establishment was £6,462. In 1899 they had forty Vice-Presidents; now they had fifty. Then they had 205 members, now 568; while their associates then numbered 2,657, and now 6,412. Those figures should be a great encouragement to other districts; they showed in what direction the work should be pursued, for Fulham was no more favoured than any other part. One point he would like to press, and that was the appointment of honorary secretaries in the districts. He hoped soon there would be no district which had not one at least, and preferably two—one lady and one gentleman. As to the total sum raised, he had purposely not attempted to give estimated figures; and he must rely on the Presidents and Secretaries in the future to get their work so forward that they could have the money in the bank in time for that meeting, since the King's Fund always sent their cheques to the various institutions before the end of the year. He hoped their total would be a large sum, and larger than last year's £18,139, so that their grant to the King's Fund might also be larger than last year's £15,000. He hoped, indeed, that this year's grant would bring their total contribution to the Fund to £80,000, representing an average of £10,000 a year for the eight years of their existence. He was glad to say their expenses had been brought on to a definite and fixed basis. There were no increases this year, except in salaries, where there was a small advance due to the great increase in the work of the districts. One of the chief difficulties of the League was the wastage that occurred owing to the numbers of people who tired of the work. If they could get together those who had joined and left, he thought they might get them to understand the real spirit of personal service in the days of health on which the League must rest to be a success, and that they would then come back. He had suggested last year that it would be a good thing to get the vice-presidents together; they had met in the Mansion House, and the meeting was a great success. He thought now the President and Vice-Presidents should look after their members and get them together at least once a year so as to bring them under the quickening influence of the League, which he felt sure was its greatest service to the community; for, whatever good was done to the hospitals by the money collected, he believed the effect on the individual from the rendering of personal service was even greater than the direct benefit to the sick and suffering.

Sir Wm. Collins, one of the Hon. Secretaries, spoke as to the work of the League, and announced that the Prince and Princess of Wales had graciously promised their patronage to a Children's Fancy Dress Ball at the Hôtel Cecil on Saturday, January 12 next.

Mr. Montagu Sharpe pleaded for more assistance to the smaller local hospitals, and this the Chairman promised should receive consideration.

Votes of thanks brought the proceedings to a close.

